# Inhaled Nitric Oxide as an Adjunct to Thrombolytic Therapy in a Patient with Submassive Pulmonary Embolism and Severe Hypoxemia

**DOI:** 10.1155/2019/5184702

**Published:** 2019-03-28

**Authors:** Omesh Toolsie, Umut Gomceli, Gilda Diaz-Fuentes

**Affiliations:** ^1^Division of Pulmonary and Critical Care Medicine BronxCare Health System, 1650 Grand Concourse, Bronx, NY 10457, USA; ^2^Icahn School of Medicine at Mount Sinai, USA; ^3^Division of Cardiology, BronxCare Health System, 1650 Grand Concourse, Bronx, NY 10457, USA

## Abstract

**Introduction:**

Inhaled nitric oxide (iNO) is a selective pulmonary vasodilator with limited indications in adults. We present a patient with hypoxemia and right ventricular dysfunction due to submassive acute pulmonary emboli where iNO was used as a bridge to thrombolysis.

**Case:**

A 29-year-old male was admitted to the intensive care unit (ICU) for alcohol intoxication complicated with aspiration pneumonia and acute respiratory failure requiring mechanical ventilation. His medical history included morbid obesity (BMI 43) and alcohol dependence syndrome. Nine days after admission, he developed severe acute hypoxia and tachycardia with arterial oxygen tension (PaO2) of 52 mmHg requiring a positive end-expiratory pressure (PEEP) of 14 cmH2O and fraction of inspired oxygen (FiO2) of 1. Chest computed tomography (CT) revealed a large embolus in the right main pulmonary artery and transthoracic echocardiogram (TTE) reported new right ventricular dilatation with decreased right ventricular function. Due to the severe hypoxemia, he was started on iNO via the breathing circuit of the ventilator at a concentration of 20 parts per million (ppm) with steady improvement in oxygenation after 1 hour with a PaO2 of 81 mmHg on the same ventilator setting. The patient was given thrombolysis with alteplase and the iNO was slowly tapered off during the subsequent four days with concomitant successful tapering of PEEP to 8 cmH2O and FiO2 of 0.45.

**Conclusion:**

Inhaled NO has been used to improve ventilation-perfusion matching and also to reduce pulmonary vascular resistance (PVR). Its effects on PVR may be useful in the setting of acute pulmonary emboli where vascular obstruction and vasoconstriction contribute to increased pulmonary arterial pressure and PVR which can present as acute right heart failure. We suggest that iNO, if available, could be considered in those patients with acute pulmonary emboli associated with significant hypoxemia as an adjunctive therapy or bridge to thrombolysis or thrombectomy.

## 1. Introduction

Nitric oxide (NO) is a naturally occurring gas, and at room temperature, it is both colorless and odorless, and when added to an inspired gas, it produces selective pulmonary vasodilation which improves pulmonary hypertension and decreases right ventricular (RV) afterload [1, 2]. This potent endogenous vasodilator can be exogenously administered via inhalation. Use of iNO in adults remains controversial. We present a patient with severe hypoxemia and acute right ventricular dysfunction due to a submassive acute pulmonary emboli where iNO was used as a bridge to diagnosis and thrombolysis.

## 2. Case

A 29-year-old male was admitted to the intensive care unit with acute respiratory failure requiring intubation in the field. The patient was found unresponsive on the floor of his apartment. His medical history included morbid obesity with a body mass index of 43 kg/m^2^ and alcohol dependence syndrome.

On initial examination he was sedated on mechanical ventilation, afebrile (98 F), hypertensive with a blood pressure of 167/69 mmHg, tachycardic with heart rate of 111 beats per minute and a respiratory rate of 20 breaths per minute synchronous to the set rate on the ventilator. Peripheral capillary oxygen saturation (SPO2) was 98% with inspired oxygen saturation (FiO2) of 0.5. Lung examination revealed rales in the mid and lower zones of the right lung field. Cardiac examination revealed tachycardia with no murmurs. Abdominal, neurologic and skin exams were unremarkable.

Relevant laboratory on admission included the following: leukocytosis (white cell count of 11.2 × 10^3^ cells/ /*μ*L), serum creatinine of 2.1 mg/dL (baseline 0.6 mg/dL), serum creatine kinase of 39, 552 units/L, serum lactic acid of 4.2 mmoles/L, and serum ethanol level of 316 mg/dL. Initial arterial blood gas analysis revealed a pH of 7.26, an arterial carbon dioxide (PCO2) level of 40.2 mmHg and oxygen level (PO2) of 108 mmHg on a FiO2 of 0.5, and positive end-expiratory pressure (PEEP) of 5. Chest X-ray (CXR) showed right upper, middle, and lower lobe infiltrates and computed tomography (CT) of the head demonstrated no acute infarcts or hemorrhage.

He was started on broad spectrum antibiotics including vancomycin, piperacillin-tazobactam, and azithromycin for pneumonia. On day 2 of admission, the patient underwent fiberoptic bronchoscopy which revealed thick mucopurulent secretions in all segments of right lung. The respiratory cultures from bronchoalveolar lavage were negative as well as initial blood and urine cultures. Transthoracic echocardiogram (TTE) on day 2 revealed an ejection fraction of 57% with normal right ventricular structure and function and normal pulmonary artery systolic pressure (PASP). The patient clinically and radiologically improved, he was awake and tolerating daily spontaneous breathing trials with FiO2 requirements of 0.4 to keep PO2 of 104.

**Figure 1:**
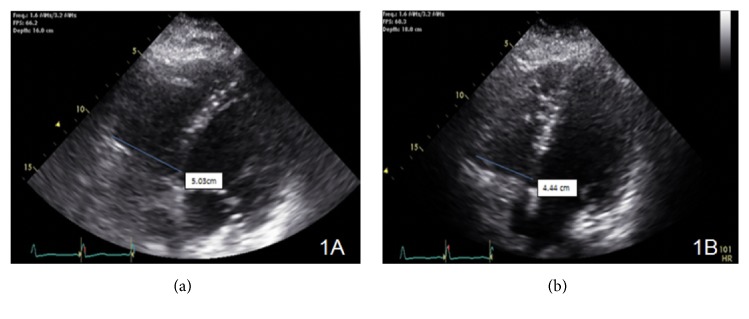
(a) Right ventricular size and function assessment was done with RV focused imaging from apical four chamber view. Initial measurement showed 5.03 cm RV basal diameter consistent with moderately dilated RV size. 2D images also showed reduced RV ejection fraction along with RV strain findings. (b) Right ventricular size and function assessment was done with RV focused imaging from apical four chamber view. RV basal diameter is measured as 4.44 cm which was consistent with mildly dilated RV size. RV ejection fraction is improved, and RV strain findings were no longer available.

On day nine of admission, he was noted to be hypoxic with a PO2 of 52 mmHg with a SPO2 of 88%. He required a PEEP of 14 cmH20 and a FiO2 of 1 to achieve a PO2 of 66 mmHg. Due to body habitus prone position was not an option. Repeated CXR did not show any new abnormalities- resolving right-side infiltrates. In addition to the subcutaneous heparin started on admission for deep venous thrombosis (DVT) prophylaxis, a therapeutic dose of low molecular weight heparin (LMWH) at 1 mg/kg was administered with a presumptive diagnosis of acute pulmonary emboli as he was too unstable for transportation to radiology. A repeat TTE showed a PASP of 54 mmHg with new moderate dilatation of the RV and moderate tricuspid regurgitation (Figure 1(a)) with a serum troponin T of 0.204 ng/mL and proBNP of 445 pg/mL. Inhaled nitric oxide (iNO) was given via the breathing circuit of the mechanical ventilator at a concentration of 20 parts per million (ppm) with improved oxygenation noted after one hour, PO2 increased to 81 mmHg with a FiO2 of 0.8. His blood pressure during this time ranged from a systolic of 117-134 mmHg and diastolic of 68–87 mmHg.

**Figure 2:**
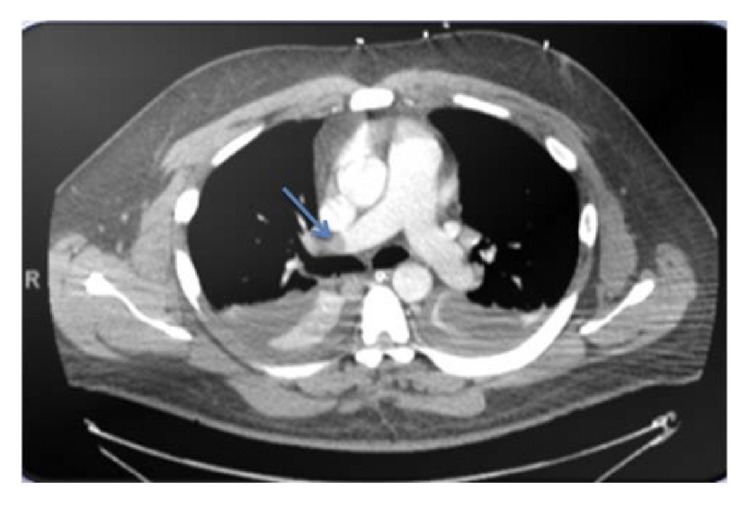
CT Chest demonstrating embolus in the right main pulmonary artery.

Chest CT angiogram revealed large embolus in the right main pulmonary artery (Figure 2). Thrombolytic therapy with alteplase at 100 mg was administered over two hours and the iNO was slowly tapered off over the next four days with concomitant tapering of PEEP to 8 cmH20 and FiO2 of 0.45. Repeat TTE three days after thrombolytic therapy demonstrated a PASP of 32 mmHg with mild dilation of the RV without signs of strain (Figure 1(b)). The patient was weaned from mechanical ventilation and later discharged to a skilled nursing facility for rehabilitation on oral apixaban for three months with outpatient follow-up provided.

## 3. Discussion

Nitric oxide (NO) is a naturally occurring gas and at room temperature it is both colorless and odorless. It is produced endogenously from L-arginine by NO synthase and when added to an inspired gas, it produces selective pulmonary vasodilation. It activates guanylate cyclase which then activates cyclic guanosine monophosphate (cGMP). Cyclic GMP stimulates cGMP-dependent protein kinase that activates myosin light chain phosphatase, which dephosphorylates myosin light chains, leading to smooth muscle relaxation. Additionally, increased intracellular cGMP inhibits calcium entry into the cell, and decreases intracellular calcium concentrations further promoting relaxation of vascular smooth muscle [3]. In the lung it acts selectively on the pulmonary vasculature because of rapid hemoglobin-mediated inactivation as it diffuses into the pulmonary capillaries [1]. With the relaxation of the pulmonary vessels, iNO reduces pulmonary vascular resistance which translates to lower pulmonary arterial pressures and reduced right ventricular (RV) afterload [2].

There are limited therapeutic indications for iNO in adults. Currently in the United States, it is licensed only for neonates with respiratory failure and persistent pulmonary hypertension. In adults, it has an established diagnostic role in vasodilator testing in patients with pulmonary arterial hypertension. iNO has been studied in patients with acute respiratory distress syndrome (ARDS) but with conflicting results. A meta-analysis by Sokol et al. reviewed five randomized control trials with a combined total of 535 patients with acute hypoxemic respiratory failure and found no significant effect in mortality with only transient improvement in oxygenation [4]. The use of Inhaled NO in adults is based on its ability to provide selective pulmonary vasodilatation in well- ventilated lung units, to improve ventilation-perfusion mismatch and subsequently to reduce the elevated pulmonary vascular resistance and pulmonary hypertension as seen in ARDS [5]. In addition, iNO has been used in RV failure associated with cardiac surgery and sickle cell disease [6].

The estimated incidence of PE in the United States in one analysis looking at patients from 1998-2006 was 112 per 100, 000 adults with a 7.8% mortality rate [7]. Several studies have found that the presence of RV dysfunction portends a worse prognosis with at least a 2-fold increased risk of death [8–12]. An experimental model looking at pulmonary vascular reserve during experimental PE noted that increased PVR was caused to a minor degree by direct mechanical obstruction and to a greater extent by vasoconstriction [13]. A sudden increase in the RV systolic pressure caused by an acute PE promotes turbulent flow across the tricuspid and pulmonic valves leading to hemolysis and release of heme (which deactivates NO) and arginase-1, reducing L- arginine levels needed for NO production. Additionally, trapped platelets release thromboxane A2 and serotonin which are potent vasoactive mediators [14–17]. Overall, reduced levels of NO in the presence of vasoactive substances further increase pulmonary vascular resistance and RV pressures in an acute PE.

Assessing RV function is essential in the risk stratification of acute PE. In our case, we highlighted acute right ventricular dilatation with moderate tricuspid regurgitation. An emerging echocardiographic marker for patients with intermediate risk PE is the ratio of tricuspid regurgitation peak gradient (TRPG) to tricuspid annulus plane systolic excursion (TAPSE) or TRPG/TAPSE. A TAPSE of < 17 mmHg is suggestive of RV systolic dysfunction but when interpreted as a ratio to TRPG, a group of Polish researchers were able to demonstrate that a TRPG/TAPSE > 4.5 was associated with 21.1% risk of PE-related death or rescue thrombolysis [18]. This echo parameter may be of added prognostic value especially when combined with conventional parameters of RV function.

There are case reports of successful use of iNO as a therapeutic agent in patients with acute pulmonary embolism (PE). Inhaled nitric oxide may have a unique role as an adjunct to existing standard of care including anticoagulation, thrombolytics and surgery in the patient with acute PE and severe hypoxia, acute RV failure or hemodynamic instability. Its role as a selective pulmonary vasodilator may reduce RV afterload without causing systemic vasodilation which is beneficial in these critically ill patients. There are case reports of successful use of iNO as a therapeutic agent in patients with acute pulmonary embolism (PE) [19]. A review of 18 patient with acute PE receiving iNO at a dose of 10 to 50 ppm for up to 6 days revealed that 14 (78%) patients had improvement in mean arterial pressure, 16 (89%) had significant improvement in oxygenation and 14 (78%) survived hospital admission. All of the patients were anticoagulated, 12 received thrombolytic therapy and 6 received either mechanical embolectomy or intra-arterial thrombolysis [20]

Our patient has similar characteristics to patients described in other reports with refractory hypoxemia and evidence of acute RV failure. The real-world challenge for the clinician practicing in a setting similar to ours, without on-site ECMO or advanced thoracic-vascular surgery, is to be able to stabilize the patient in order for safe transportation for diagnostic or therapeutic purposes. At our institution, iNO is available for use in neonates. We extrapolated data from pediatrics and from reported use in similar cases of intermediate risk PE with RV strain in our decision to use iNO and at what dose. There has been significant interest regarding the potential therapeutic use of iNO in PE with the first phase II double-blinded, randomized trial of iNO to treat acute PE recently completed by Kline et al. with the final analysis to be reported [21].

Inhaled epoprostenol (iEPO) is another inhaled pulmonary vasodilator that like iNO has been used in patients with ARDS and refractory hypoxemia. It functions like iNO, causing selective pulmonary vasodilation when added to an inspired gas. Webb et al. described a case in 1996 where its use in a patient with massive PE resulted in a transient improvement in mean pulmonary arterial pressures but did not alter the clinical deterioration of the described patient [22]. While there have been no studies assessing the role of iEPO in acute submassive PE, the use of systemic epoprostenol was studied by Kooter et al. who conducted a randomized controlled trial comparing its use to placebo in patients with a diagnosis of acute PE and right ventricular overload. The researchers were unable to find any significant improvement in right ventricular dilatation or other objective markers of right ventricular overload with the use of systemic epoprostenol [23]. While the patients in this study did not develop hypotension, the use of a systemic vasodilator should be used with caution in this setting as it may precipitate hemodynamic collapse in patients with acute RV dysfunction. iEPO is not available at our institution and thus not considered in our patient

## 4. Conclusion

Our patient highlights the challenges in the evaluation and management of critically ill patients with acute pulmonary emboli and severe hypoxia and right-side failure.The use of iNO likely plays a role in the initial stabilization of these patients allowing time for more definitive therapy and may even decrease the need for more invasive therapeutic modalities. These potential benefits will need to be assessed in large prospective trials.

## Abbreviations

RV: Right ventricle
ICU: Intensive care unit
PEEP: Positive end-expiratory pressure
CT: Computed tomography
TTE: Transthoracic echocardiogram
iNO: Inhaled nitric oxide
PVR: Pulmonary vascular resistance
FiO2: Fraction of inspired oxygen
CXR: Chest X-ray
PASP: Pulmonary artery systolic pressure
PE: Pulmonary embolism.
